# Anastomotic Strictures after Esophageal Atresia Repair: Timing of Dilatation during the First Two Postoperative Years

**DOI:** 10.1055/s-0038-1646950

**Published:** 2018-05-07

**Authors:** Martin Salö, Pernilla Stenström, Magnus Anderberg, Einar Arnbjörnsson

**Affiliations:** 1 Division of Pediatric Surgery, Department of Clinical Sciences, Pediatrics, Lund University, and Skåne University Hospital, Lund, Sweden

**Keywords:** esophageal atresia, anastomotic stricture, balloon dilatation timing, infants

## Abstract

**Background** We determined time frames for dilatation of anastomotic strictures (ASs) occurring during the first 2 years after esophageal atresia (EA) repair.

**Methods** A retrospective study was conducted on children with EA (Gross type C) who underwent direct repair between January 2008 and March 2015 at a single tertiary center of pediatric surgery. Endoscopic signs of stricture were indications for dilatation because the endoscopy provides more reliable information than X-ray imagining methods.

**Results** Among our cohort of 49 children with EA, 19 (39%) required at least one esophageal dilatation. All children required initial dilatation within the first year of life and none was older than 1 year during initial dilatation ( *p* < 0.01). A median of three dilatations (range: 1–13) took place per patient, with 87% performed during the first postoperative year. The timing of initial dilatation in the first year (< 6 months, 14/19 [74%] vs. 6–12 months, 5/19 [26%]) was predictive of the need for dilatation beyond the first year (9/14 [64%] vs. 0/5 [0%]; *p* = 0.03) but not of more numerous dilatations (median, 3 vs. 1; *p* = 0.07).

**Conclusion** The need for dilatation within 6 months postoperatively predicts the need for dilatation after 1 year, but it does not indicate the number of dilatations that will be needed.

Anastomotic stricture (AS) occurs in 9 to 79% newborns after esophageal atresia (EA) repair. 1 2 3 4 5 6 The timing of dilatation procedures (i.e., when and how often) throughout the postoperative period in a long-term follow-up has been addressed in a previous report which showed that a great majority of dilations are performed within the child's first 2 years in life. 7 Further insight in the course during the first two postoperative years would be beneficial because guardians of affected neonates deserve timely counseling on the risk of AS and the potential need for an intervention.

The aim of this study was to determine when most dilatations are needed for AS relative to the patient age after EA repair to provide information that can be used in counseling. The main question was whether children who need dilatation within the first 6 months also require dilatation after the first year or if such early need of dilatation predicts need for numerous dilatations.

## Methods

### Study Design

Data were collected at a tertiary center of pediatric surgery. All study subjects had undergone primary anastomosis of EA with distal tracheoesophageal fistula (Gross type C), and hence, there were no children with a long gap EA. The surgeries were performed between January 2008 and March 2015. The information was retrospectively gathered from charts and from 2011 from a prospectively acquired database. Results of EA repair in this cohort have been reported previously. 7 8 9

Primary study outcomes were timing and frequency of dilatations performed for AS during postoperative monitoring of each patient up to the latest counseling session at our department or at least till the age of 2 years. The 2-year period was chosen because majority AS dilatations occur during that period. 7 8 9 All children had prophylaxis with proton pump inhibitors (PPIs) during 3 to 12 months postoperatively. 7 8 9

A stricture was defined as narrowing of the esophagus. These were identifiable on X-rays through the use of contrast. Contrast esophagograms were routinely performed at 1 to 3, 6 to 8, and 12 months postoperatively or upon clinical suspicion of a stricture (i.e., dysphagia, difficulty in swallowing, and/or repeated vomiting). The final diagnosis of AS was verified by esophagoscopy. Endoscopy provided more reliable information than X-ray imagining methods. In our practice, based on our experience and reports from the literature, 1 we performed repeated dilatation when needed within 2 to 3 weeks. Waiting for a longer period may lead to narrower strictures resulting in more symptoms in the child. Thus, the children were admitted because of the symptoms, examined by contrast esophagogram, endoscopy, and if a stricture was identified, further regular dilatation was performed until the AS vanished.

### The Dilatation Technique

All dilatations were performed with patients under general intubation anesthesia and using fluoroscopy. Endoscopic dilatation involved the use of controlled radial European balloon dilators (Boston Scientific, Watertown, MA) and a video endoscope (GIF-XP160; Olympus Corp, Tokyo, Japan).

Dilatation or calibration was performed no earlier than 3 weeks after initial EA repair and was repeated at intervals of 2 to 3 weeks guided by the symptoms reported by parents or for stricture resolution on esophagograms. The balloon size varied from 5 to 20 mm. The size of the balloon was decided by the size of the child's thumb. 10 The duration of dilatation with an inflated balloon in the esophageal stricture was 3 minutes according to a local routine.

Dilatation was defined as widening of the AS diameter as much as the caliber of a child's thumb. 10 Balloons used for dilatation were inflated with contrast during fluoroscopic imaging using 3 to 6 atmospheres of pressure 1 as needed. If balloon contours were reduced by AS, the procedure qualified as a dilatation. If not, it was viewed as a calibration.

No child underwent stricture resection and primary esophageal anastomosis. No motility agents were used during the period studied.

### Statistical Considerations

Prior data indicated that the probability of AS after EA during the first year of life is 0.5. 1 2 3 4 5 6 7 8 9 10 11 The correlation coefficient for exposure between matched test and control subjects is 0.1. If the true odds ratio (OR) for developing AS during the first year (relative to controls older than 1 year) is 0.1, a total of 19 test subjects, each with a single-matched control, is needed for rejecting the null hypothesis that this OR = 1 at a probability (power) of 0.8. The probability of a type I error associated with this test of the null hypothesis is 0.05.

Statistical methods used were the Fisher's exact probability test, Freeman–Halton extension of Fisher's exact probability test for two-row by three-column contingency table, and the Mann–Whitney *U* test.

### Ethical Approval and Consent to Participate

The study procedures were followed in accordance with the revised Declaration of Helsinki (1964) and the Good Clinical Practice guidelines. The study was approved by the Regional Ethical Review Board (registration number 2010/49). The included children were registered after consent according to the regional demands on quality register, number 01481271007173. All data were coded and rendered anonymous. Administrative permissions from the hospital institution were received for access of medical records.

## Results

A total of 50 children underwent surgery for EA Gross type C during the study period. The mean ± standard deviation (SD) age at EA reconstruction was 1 ± 1 (median 1, range: 0–3) day.

The main postoperative complications were leakage in the anastomosis in five (10%) neonates, of whom two did and three did not have AS. Tracheomalacia requiring operative intervention was observed only in one (2%) infant. One infant died of severe cardiac malformation within 4 weeks and was excluded.

Thus, 49 children qualified for further analysis. The mean gestational age was 38 ± 3 months and 28 (57%) were full-term, with a mean birth weight of 3 ± 1 kg. Among the 49 infants included in the study, 19 (39%; 16 males and 3 females) required dilatations for AS. The follow-up time was set to 2 years, which was the endpoint of the study.

Dilatations were performed at ages ranging from 5 weeks (time of first dilatation) to 2 years. All 19 children required their first esophageal dilatation early (during the first 6 months, 14/19) or late (> 6–12 months, 5/19) in the first year of life. No child required their first dilatation after the age of 1 year. No dilatation-related perforations, requiring operative interventions, were recorded in this study population. The 19 children required 71 dilatations in total. The mean ± SD number of dilatations per patient undergoing at least one dilatation procedure was 4 ± 3, and the median number of dilatations was 3 (range: 1–13; Table 1 ). In total, 53% (38/71) dilatations were performed within the first 6 months postoperatively. The number of children requiring dilatation and the frequency of such dilatations significantly declined over time ( Table 1 ). The number of calibrations performed is summarized in Table 1 .

**Table 1:** Summary of dilatations in newborns ( *n* = 49) with reconstructed EA and the subset ( *n* = 19) requiring dilatation because of AS during the first two postoperative years

		*p* -Value
Total number of dilatations needed ( *N* )	71	
Dilatation per child who underwent dilatation		
- Mean ± SD	4 ± 3	
- Median (range)	3 (1–13)	
Age at initial dilatation		
6 mo	14 (74%)	< 0.01*
> 6–12 mo	5 (26%)	
> 1 y	0 (0%)	
Periodic dilatations, *n* (%)		
< 6 mo	38 (53)	< 0.01*
6–12 mo	24 (34)	
> 1 y	9 (13)	
Number of dilatation in each child		
With initial dilatation at < 6 mo	3 (1–13)	0.07**
With initial dilatation at > 6–12 mo	1 (1–7)	
Number of calibrations performed	21	

Abbreviations: AS, anastomotic stricture; EA, esophageal atresia; SD, standard deviation.

Note: Values presented as the number and absolute percentage of patients, *n* (%), and as mean ± SD and median (range, min–max).

*Freeman–Halton extension of Fisher's exact probability test for two-row by three-column contingency table.

**Mann–Whitney *U* test ( *Z* score is 1.80535).

During the postoperative period, the percentage of children requiring dilatation before and after the age of 1 year significantly differed on the basis of the timing of initial intervention within the first year (< 6 [36%] and 6–12 [0%] months, respectively; *p* < 0.03). The need for initial early dilatation (< 6 months postoperatively) compared with initial later dilatations (6–12 months postoperatively) did not predict the numbers of dilatations required (median, 3 [range: 1–13] and 1 [range: 1–7], respectively; *p* = 0.07).

There was no significant correlation among esophageal stricture with anastomotic leakage, gap between the esophageal end, gestational age, gestational weight, operation time, operation duration, surgeon, suture material (size, number, and nature), gastroesophageal reflux, infections, postoperative ventilator support, and associated anomaly.

## Discussion

The results showed that in this cohort of children with reconstructed EA Gross C, dilatation of AS was needed in 39% children within the first two postoperative years. Dilatation within the first 6 months postoperatively predicted the need for dilatations also when the child was older than 1 year but early dilatation was not associated with more numerous dilatations. The main information provided by this study was that if patients were dilated early, within 6 months, they would need prolonged but not significantly more dilatations than those who may need a dilatation after 6 months postoperatively ( Table 1 ).

There was no significant correlation between the esophageal stricture and possible reasons for it, such as anastomotic leakage, length of the gap between the esophageal ends, gestational age, gestational weight, timing of the operation, duration of the operation, surgeon, suture material (size, number, and nature), gastroesophageal reflux, infections, postoperative ventilator support, and associated anomaly. These findings may be due to the small number of included patients.

All children had prophylaxis with PPIs during 3 to 12 months after EA repair. During the period studied, PPI was considered necessary to reduce acid gastroesophageal reflux and thereby reduce the frequency of AS. This idea has not yet been supported by any scientific evidences. Other investigators have aimed to assess the efficacy of prophylactic antireflux medication in reducing stricture formation and concluded that this appeared ineffective. 2

The frequency of dilatations in this study group did not differ significantly from that reported previously. 1 2 3 4 5 6 7 8 9 10 11 12 The small number of children included is a limitation of the study. Compared with our previous report, 2 this study focused on only the first two postoperative years after EA repair. The intention was to collect further details on the timing of dilatations of AS after EA reconstruction. This information is important when planning for health care of children with EA and for providing to their guardians.

Adjuncts to dilatation, such as local steroid injection, topical mitomycin C application, and esophageal stents, were not used during the study interval and consequently did not confound the results. Currently, there is insufficient evidence to promote one adjunctive therapy over another. 1

Compared with our previous report, 2 this study focused on only the first two postoperative years after EA repair which is of most concern for a majority of guardians and for planning the medical care. In most children requiring dilatation, 14 of 19, the procedures were initiated, in the first half year of life versus the second half year of live in 5 of 19. This has relevance when counseling guardians. In addition, if a child did not need dilatation for AS by the age of 1 year, it turned out to be unlikely that any dilatation at all would be necessary during the second year.

Adequate data pertaining to the frequency and timing of postoperative AS and the need for dilatation is important for parental counseling and planning of long-term postoperative care in patients with EA. It is imperative that parents of such patients be informed of the potential for esophageal narrowing over time.

The study has several limitations. First, the numbers of patients are limited. It is premature to determine outcomes at 2 years of age when counseling parents or guardians of children with EA. A report of outcomes at 5 years of age would better address the aim of that question. Furthermore, the question of durability of dilatations in achieving long-term patency of the esophageal anastomosis with stricture was not clearly answered by this study. Because some patients may again experience this problem as adults, 2 referral to specialists for adult care is advised. Further investigation of dilatation requirements during adulthood is warranted.

Another limitation is that deciding if there is a stricture or not could be vague. As indicated in our methods, we performed routine contrast esophagograms after EA repair in patients with and without symptoms. However, dilatation was performed based on symptoms and findings of upper endoscopy. If we found a radiologic stricture on esophagography in a patient without clinical findings, dilatations were not performed. In all 19 patients who required dilatation as well as in those not requiring dilatation, an anastomosis in the esophagus could be visualized on the esophagogram and might have been interpreted as a stricture on a routine esophagogram. Thus, the findings on the routine esophagogram only cannot be used to decide which children to select for esophageal stricture dilatation. Furthermore, findings on esophagogram could not provide information on deciding if these patients required early and repeated intervention within the 2-year period studied.

Calibrations are also a part of the dilatation procedures. To avoid inadequate balloon dilatation with a too small diameter balloon, the diameter of the child's thumb has been used at our center since it was first reported. 10 Thus, the question of a subjective character which is the size of the balloon is solved. In our practice, the use of the diameter of the child's thumb is easy and clear and makes the dilatation procedure consequent and correlated with each child's thumb. By correlating with the thumb, the use of a too small or too large balloon is avoided in individual children of varying size during the time of growing up.

The strength of this study was that perioperative management of all our children took place at the same center with continuous evaluation of outcomes. However, the fact that data were compiled prospectively and retrospectively at different time periods was a limitation. In addition, durations of follow-up were short, with no monitoring into adulthood. A weakness of this study was that the small patient cohort was split into still smaller groups. We do not refute that a larger sample size could have resulted in significance regarding whether early dilatation also predicts a higher number of dilatations during childhood. Further weakness of this study was that there was a lack of documentation on the degree of ASs. This was due to a lack of information in retrospective data collection. Instead of documenting the degree of ASs, dilatations were performed up to the size of the child's thumb, according to the method followed in previous reports. 10

## Conclusion

The need for dilatation within 6 months and 1 year postoperatively predicts the need for dilatation also after 1 year, but it does not indicate the number of dilatations that will be needed.
